# Co-creation of a digital tool for the empowerment of parents of children with physical disabilities

**DOI:** 10.1186/s40900-017-0079-6

**Published:** 2017-12-11

**Authors:** M. W. Alsem, K. M. van Meeteren, M. Verhoef, M. J. W. M. Schmitz, M. J. Jongmans, J. M. A. Meily-Visser, M. Ketelaar

**Affiliations:** 1 Center of Excellence for Rehabilitation Medicine, Brain Center Rudolf Magnus, University Medical Center Utrecht and De Hoogstraat Rehabilitation, Rembrandtkade 10, 3583 TM Utrecht, the Netherlands; 2 Department of Rehabilitation Medicine, Academic Medical Centre, Amsterdam, the Netherlands; 3 Zuyd University of Applied Science, Research Centre for Data Intelligence, ICT Faculty, Heerlen, the Netherlands; 4 Department of Child, Family & Education Studies, Faculty of Social and Behavioural Sciences, Utrecht University, Utrecht, the Netherlands; 5 Department of Neonatology, Wilhelmina Children’s hospital, University Medical Center Utrecht, Utrecht, the Netherlands

**Keywords:** Co-creation, Parents, Empowerment, Information, Internet, Health literacy

## Abstract

**Plain English summary:**

Parents of children with physical disabilities do a lot to support their child in daily life. In doing this they are faced with many challenges. These parents have a wide range of unmet needs, especially for information, on different topics. It is sometimes hard for them to get the right information at the right moment, and to ask the right questions to physicians and other healthcare professionals. In order to develop a digital tool to help parents formulate questions and find information, we thought it would be crucial to work together in a process of co-creation with parents, researchers, IT-specialists and healthcare professionals. In close collaboration with them we developed a tool that aims to help parents ask questions, find information and take a more leading role in consultations with healthcare professionals, called the WWW-roadmap (WWW-wijzer in Dutch).

In two groups of parents (one group with and one group without experience of using the tool), we will study the effects of using this tool, on consultations with physicians. We expect that using the tool will result in better empowerment, satisfaction and family-centred care.

**Abstract:**

**Background:**

Parents of children with physical disabilities do much to support their child in daily life. In doing so, they are faced with many challenges. These parents have a wide range of unmet needs, especially for information, on various topics. Getting timely and reliable information is very difficult for parents, whereas being informed is a major requirement for the process of empowerment and shared decision-making. This paper describes the development of a digital tool to support parents in this process. During its development, working together with parents was crucial to address relevant topics and design a user-centred intervention.

**Methods:**

In co-creation with parents, healthcare professionals, IT-professionals and researchers, a digital tool was developed, the ‘WWW-roadmap’ [‘WWW-wijzer’ in Dutch]. This digital tool aims to enable parents to explore their questions (What do I want to know?), help in their search for information (Where can I find the information I need), and refer to appropriate professionals (Who can assist me further?).

During the process, we got extensive feedback from a parent panel consisting of parents of children with physical disabilities, enabling us to create the tool ‘with’ rather than ‘for’ them. This led to a user-friendly and problem-driven tool.

**Discussion:**

The WWW-roadmap can function as a tool to help parents formulate their questions, search for information and thus prepare for consultations with healthcare professionals, and to facilitate parental empowerment and shared-decision making by parent and professional. Effects of using the WWW-roadmap on consultations with professionals will be studied in the future.

## Background/Introduction

### Family-centred care and the role of parents in paediatric rehabilitation and research

In paediatric rehabilitation, interventions mainly focus on improving the functioning and participation of children with disabilities as defined in the International Classification of Functioning, Disability and Health, Child and Youth version (ICF-CY) [1]. The close interaction between the functioning of children and that of their families has been widely acknowledged, [2–5] but shifting focus from child-centred to family-centred principles in clinical practice still seems difficult [5–7]. Shifting focus towards the family as a whole, and toward family engagement, parent partnership and other family-centred principles, is a process in which the respective roles of parents and professionals have to be jointly defined [8].

In advocating these family-centred principles in research and clinical practice, collaboration between parents, researchers and clinicians is self-evident. Parents are usually the experts on their child and family, and should therefore be involved and engaged in designing and evaluating tools to enhance family-centred care and family functioning. Only then can one be sure to create a solid base of support for such interventions. We therefore invited parents, clinicians and researchers to join in designing and examining an intervention to provide parents and physicians with a tool that parents can use to search and find information and determine their own preferred role in the care process.

This paper describes the collaborative design and development of a digital tool, the WWW-roadmap. Parents were involved in all phases of the project.

### The role of information in (family) empowerment

In order to make paediatric rehabilitation care more family-centred, parents and families of children with disabilities should be able to express their family needs, take part in decision-making and be provided with opportunities and abilities to do so.

Empowerment is defined as the ‘*… acquisition of motivation (self-awareness and attitude through engagement) and ability (skills and knowledge through enablement) that patients might use to be involved or participate in decision-making, thus creating an opportunity for higher levels of power in their relationship with professionals’*. [9, pg 390] Supporting and enhancing (family) empowerment creates a basis for involving parents and family in the care process, and thus makes the care more family-centred. However, concrete tools to help shift the focus towards the family as a whole are currently lacking.

An important factor in the process of empowerment is the acquisition of knowledge. [9] Moreover, access to health information is critical to making decisions [10].

In previous research, parents expressed a variety of needs for information [11–15]. These needs included information on their child’s condition, prognostic information about the child’s development, but also about possible treatments and assistance. Information has been found to have a positive effect as a coping strategy and to make parents feel more in control [16, 17].

The ‘*… cognitive and social skills, which determine the motivation and ability of individuals to gain access to, understand and use information in ways which promote and maintain good health*’ form a concept that is known as ‘health literacy’ [18, 19 pg 6]. These skills could be used for activities such as searching for information and asking questions, which in turn are a prerequisite for increased participation in the consultation between parents and professionals and for parental empowerment.

Better health literacy contributes to better general health [18–20] and parental health literacy favourably affects children’s health outcomes, [21, 22] while low levels of health literacy have a negative impact on rehabilitation interventions [23].

**Fig. 1:**
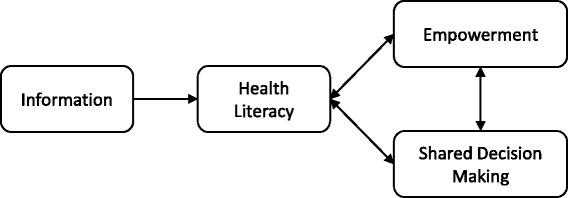
The role of information

Factors included in health literacy, i.e., finding, understanding and using (written) information, are thus important determinants of empowerment [24] and (shared) decision making (see Fig. 1) [25, 26].

### What is the problem?

Both parents and healthcare professionals find it difficult to shift focus towards the family and towards collaborative, shared decision making. Determining their role in the process is difficult for parents as well as professionals [5, 8, 26]. For parents it is also difficult to assess and express their family needs as well as to make plans to meet these needs [13, 14, 27–29]. Additionally, physicians report various barriers to addressing non-medical topics, for instance insufficient knowledge of psychosocial factors and lack of time to address these issues [30, 31].

Whereas it is evident that information is important for empowerment [24] and a key component of family-centred services (FCS), [32] we know that parents of children with physical disabilities report being unhappy about the lack of information [14, 16, 26, 33–35].

### Closing the information gap

On the supply side, essential aspects include the timely provision of the right information, and the presentation of information in such a way that it matches parental questions and preferences [16, 24, 36]. On the demand side, it is important that parents can formulate and express their information needs. Information needs differ between parents [11, 37, 38] and change over time, [28] and thus call for a tailored approach [26, 39, 40].

Parents use different sources to find information. In the past, doctors were the main source of health information, and although times are changing and parents now use other sources, such as the Internet, [41, 42] many parents still prefer to receive medical information verbally from a doctor [43–45].

In recent decades, the Internet has become more and more integrated in daily life, [46] and health-related information is frequently searched for [10, 44, 47, 48]. However, information found on the Internet is not routinely discussed between physicians and parents [10, 44, 47]. Additionally, because the quality and reliability of information on the Internet are difficult to assess, [49] parents find it hard to determine the value of information found on the Internet [50].

Information provision and assessment of current needs are very important to provide parents with the opportunities, abilities and motivation to adopt a more ‘empowered’ role in consultations with healthcare professionals (physicians or other professionals).

Our project aims to develop and evaluate a digital tool to help parents assess their family needs and formulate information questions, to search and find information, to ask questions and thus to take a part in decision-making. This paper describes the development of this tool, which took place in a user-centred context, in close collaboration with parents, clinicians and researchers. After the tool has been developed, we are planning to study the effects of using it on parental empowerment and satisfaction about consultations with a paediatric rehabilitation physician.

## Developing the WWW-roadmap

In order to support parents and professionals in dealing with the above problems, we developed the WWW-roadmap (‘WWW-wijzer’ in Dutch). The three W’s stand for What, Where and Who, defining the three main goals of the WWW-roadmap: What do I want to know? Where can I find answers to my questions? Who can assist me further?

### The first steps

The WWW-roadmap was developed in an iterative co-creation process with parents, healthcare professionals and researchers. The researchers started by identifying possible family (information) needs of parents of children with physical disabilities; the process of collaborating with parents in these first steps has been described earlier [15]. A literature review was conducted to identify possible needs [51]. The resulting list was then reviewed and supplemented by 50 parents who were interviewed on this topic. In addition, 30 healthcare professionals and 5 parents in panel meetings were asked to reflect on the list [15]. This resulted in a list of 189 possible family needs that was printed as a booklet; the Family Needs Inventory (FNI) [15]. Parents could tick currently unmet needs, and take this booklet with them to consultations as a question prompt list. During the development of the FNI, and in a small pilot study in the outpatient department for paediatric rehabilitation in De Hoogstraat Revalidatie (Utrecht, the Netherlands), we asked both parents and healthcare professionals for feedback.

Strong points of the booklet that were mentioned were the list of suggested needs and topics provided to parents, the possibility to prepare for consultations and its possible use as a guidance for topics during the consultation. Disadvantages brought up by parents and professionals were the length of the booklet (15 pages), the possible burden on parents in terms of confrontation and the time investment during consultations. We concluded from the parents’ comments that the paper version of the FNI was not feasible as a clinical tool, and a digital tool was needed to enhance the functionality of the FNI.

### Collaboration and co-creation

In order to further develop a tool that would fit the purpose of helping parents assess their family needs and formulate information questions, search and find information, ask questions and thus take part in decision-making, we invited researchers, healthcare professionals, parents and ICT specialists to collaborate.

The parents involved in this study were recruited in various ways. At the beginning of the project, we started with a panel of parents who had been involved in preceding projects that gave rise to developing the WWW-roadmap. The panel started with 16 parents who had been interviewed in an earlier study on parental information needs and parents who had been involved in the FNI project. These parents were asked to provide feedback on subsequent steps in the development process. One of them, a mother of a child with cerebral palsy (KM), was included in the project group to coordinate the panel. In addition, we asked parents to cooperate through a parent organisation (BOSK), and via Facebook and Twitter. We also asked parents in the waiting room of the outpatient department of our rehabilitation centre for their opinion. Thus, a diverse group of over 30 parents was involved in the parent panel of this project. In addition, parents on the panel used their social network to ask others to participate.

The parent panel was asked for feedback in all stages of the project. Parents were asked for their opinion and input via e-mail and Facebook, but could also provide feedback on early versions of the tool in face-to-face conversations. Parents were asked for feedback in all stages, but were not obliged to do so. Some calls yielded no reactions, while others produced much feedback.

Reactions and feedback from the panel were considered essential in creating a tool designed to be used by parents. When in doubt, feedback was discussed with the project group, where a parent representative was also present, until consensus was reached. However, parental opinions were decisive especially in the process of development, as they were part of the target group that would be using the tool.

### Creating the digital tool

In the first step, content was added to the list of topics. This process was led by the mother who was part of the project group (KM). Together with the members of the parent panel and healthcare professionals (physicians, physical therapists, social worker, psychologists), we collected information on the topics and links to reliable websites.

The information consisted of short sections of text with links to reliable websites on the Internet. The goal of the tool was not to provide a comprehensive information base, but to offer directions to reliable information and professionals. The content was thoroughly reviewed by professionals and parents to ensure that the information was reliable and understandable. If there was no consensus about the reliability of the information, or about what would be appropriate information, the text was discussed in the project group until consensus was reached. For instance, many potentially interesting topics and therapies were raised on the topic of ‘medical information’. For the sake of readability of the paragraphs and the agreed purpose of the tool, we decided not to present information on specific therapy options however, but to offer general information and hyperlinks to reliable websites. In order to give users the opportunity to correct and supplement information, we decided to implement a feedback option.

Together with the IT-specialists, we used a user-centred design approach in developing the digital tool [52–54]. Thus, early mock-ups of the tool (‘paper prototypes’) were presented to the parent panel, healthcare professionals and other interested persons. This resulted in feedback from 15 parents, 5 healthcare professionals and 5 clinical researchers. This feedback was then used to further improve the usability and features of the tool, and a first ‘working’ prototype was developed. This first prototype was presented to six parents of the parent panel, and six others who had shown interest in the project on Facebook and other social media. Parents were asked to use the WWW-roadmap and express (i.e. ‘think aloud’ about) their views on usability, features and the information provided in the tool. This procedure again resulted in very important feedback that was discussed in the project group and processed in the next version of the tool. This feedback concerned usability as well as content and functions. For instance, the instructions and the texts on needs were shortened and made clearer, the overall layout was changed, and the glossary was given a more prominent place. All parents endorsed the purpose of the tool and said they would like to use the tool when it had been finished.

### Functions of the WWW-roadmap

The WWW-roadmap aims to support parents of children with physical disabilities in exploring their questions and information needs. It consists of a list of possible questions on a broad range of topics, varying from therapy options to supporting other family members. The tool can be searched using keywords and browsed on categories. The WWW-roadmap also suggests other topics that might be of interest. For instance, clicking on a topic regarding transportation will bring up the suggestion to look at possible fees and reimbursements for transportation.

After selecting topics of interest, parents can read the information provided by the WWW-roadmap and are referred to reliable websites for additional information. In addition, parents are directed to healthcare professionals who might help them answer their questions.

Any remaining questions can be placed on a question prompt list that can be taken along to a consultation with a healthcare professional. Using question prompt lists is a good way to enhance patient participation and communication between patient and professional [55, 56]. The tool also includes a glossary to help users understand the medical and other terminology.

In summary, the WWW-roadmap consists of a combination of assessment of information needs, information provision and a question prompt list. It can be used by parents at any time, and can function as a means of preparing for a consultation with a healthcare professional.

### Evaluation study

Our evaluation will determine the value of the tool by assessing the differences between parents who have used the WWW-roadmap prior to a consultation with a rehabilitation physician and parents who have not. We will study whether the WWW-roadmap supports parents in exploring their questions and in searching and finding information, and whether this enhances their empowerment.

In addition, effects on the consultations with physicians will be studied. We will examine if:

- using the WWW-roadmap prior to the consultation with a physician supports the empowerment of parents of children with physical disabilities;
- the WWW-roadmap supports parents in exploring their questions and finding information on the Internet;
- parents who have used the WWW-roadmap are able to take a more leading role in addressing topics to be discussed during the consultation with a physician and if the tool helps to increase parental self-efficacy and parent and physician satisfaction with the consultation;
- the WWW-roadmap facilitates family-centred services.

### Methods and analysis of the evaluation study

**Fig. 2:**
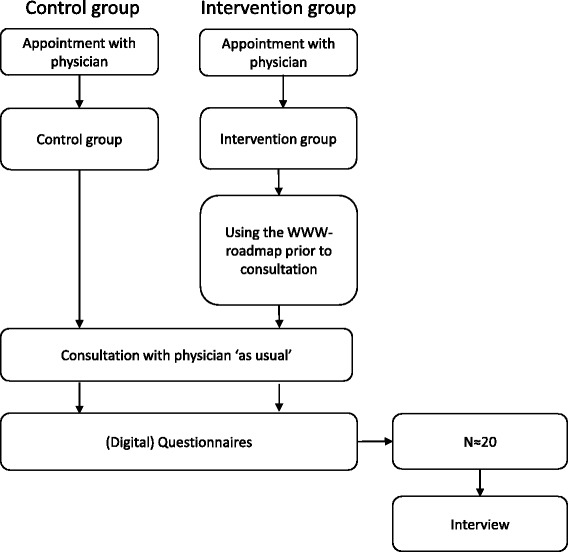
Flowchart

To study possible effects and mechanisms, we have designed a multi-centre evaluation study, which will be conducted in ten Dutch rehabilitation teams in five different settings. Settings range from rehabilitation centres to university hospitals and special needs schools. Together with the Dutch Association of People with Disabilities and their Parents (BOSK) we designed this study and determined its outcome parameters. In two cohorts of parents of children with disabilies, we will examine the effects of using the WWW-roadmap on consultations with a rehabilitation physician. Parents in the control cohort will receive care as usual, while parents of the intervention cohort will be asked to use the WWW-roadmap before their visit. Both cohorts will be asked to fill in a questionnaire afterwards, consisting of standardised questionnaires on Family Empowerment (Family Empowerment Scale, FES [57–59]), patient and physician satisfaction (Patient Satisfaction Questionnaire, PSQ [60, 61]), self-efficacy (Perceived Efficacy in Patient–Physician Interactions (PEPPI-5) [62, 63]) and family-centredness of services (Measure of Processes of Care, MPOC-56 [64, 65] and Measure of Processes of Care Service Providers, MPOC-SP [66]). For the intervention cohort, we developed a short 15-item questionnaire of additional questions for parents and professionals to assess their general experiences with the WWW-roadmap and its use. These questions focus on practical experiences (e.g. ‘The WWW-roadmap helped me prepare for the consultation’) and the use of the WWW-roadmap (e.g. ‘The WWW-roadmap is easy to use’). About 20 parents in the intervention cohort will be interviewed to qualitatively assess the effects and underlying mechanisms (Fig. 2).

## Discussion

In collaboration with parents, healthcare professionals, IT professionals and researchers we designed a tool to help parents formulate their questions and search for information, with the aim of enhancing parent empowerment. In view of the different perspectives of parents and IT and healthcare professionals, collaboration was indispensable in order to create a complete and clinically usable tool. Having a parent of a child with a disability taking part as a member of the project team was very valuable as it provided us with first-hand low-threshold comments and ideas about functionalities and content. This parent also formed the link between the researchers and the parent panel. Together with our parent panel, and in close collaboration with the Dutch Association of People with Disabilities and their Parents (BOSK), this resulted in a true co-creation process for the tool, ensuring a support base that should facilitate implementation of the tool in clinical practice.

The process of involving parents was not always easy. The process of decision-making during the development had not been clearly described beforehand. For instance, although parents had a leading role in the process of development, researchers still led the way in designing the evaluation study. We realised that the various stages of the project involved different levels of involvement. Prior definition of their respective roles in the different phases of the project could have clarified the expected roles of researchers and parents, and facilitated more active involvement in these phases. In future projects, we intend to discuss and describe these roles in all stages of development and research beforehand.

In the upcoming study described above, we will evaluate the effects and experiences of using the tool as a preparation for consultations with rehabilitation physicians. These effects will be measured quantitatively in terms of empowerment, self-efficacy and perceived family-centeredness among parents as well as physicians. As far as we are aware, this will be the first study to evaluate a tool that uses a combination of exploration of questions, information provision and a question prompt list to help empower parents.

### Limitations of the tool

The WWW-roadmap is a digital tool, mostly in Dutch. This reduces its usability, as not all parents have access to the Internet, and parents with insufficient command of the Dutch language (e.g., immigrants) might have difficulty using the tool, despite our effort to create a glossary to explain difficult words and medical jargon. Translating the tool might be an option in the future, but assessing needs and preferences of these specific groups of parents is needed in order to find out whether using a digital tool is preferred by them, or that other types of tools and information are more suitable. Not only linguistic aspects, but also cultural aspects could play a role in parental needs and empowerment. Also, the WWW-roadmap does not substitute information provision by professionals, but could merely serve as an addition. Professionals should always check if information is understood and applicable to the specific situation of the parents. Since family needs and health literacy differ between parents, a single tool cannot meet the whole spectrum of parental needs to support the process of empowerment. Professionals and parents should jointly assess the best way to support families, using available tools and interventions. The WWW-roadmap is one of the tools to support in health literacy and empowerment and could be used by a large group of parents.

The WWW-roadmap can function as a tool to help parents formulate their questions, search for information and thus prepare for consultations with healthcare professionals, and can facilitate empowerment and shared decision-making. In the upcoming evaluation study, the WWW-roadmap will be used prior to a consultation with a physician. After the study, the WWW-roadmap will be made generally available, so parents can use it as they please.

During the project, we involved parents who were interested and wanted to help with the further development of the WWW-roadmap. Our panel of involved parents started with 16 members, and increased at every step of the tool design process and the study. Until now, this panel has been queried at the initiative of the researchers. In order to keep the WWW-roadmap accessible and up-to-date, active involvement of all parties is needed to assess barriers and facilitators for optimum use and benefit of the WWW-roadmap. In fact, parental involvement is the only way in which future accessibility can be assured. This will involve new challenges and projects that will help shift the focus towards involvement of parents as true collaborators in innovation and research.

## Abbreviations

FCS: Family Centred Services
FES: Family Empowerment Scale
FNI: Family Needs Inventory
ICF-CY: International Classification of Functioning, Disability and Health, Child and Youth version
MPOC: Measure of Processes of Care
PEPPI-5: Perceived Efficacy in Patient–Physician Interactions
PSQ: Patient satisfaction Questionnaire
